# Applying the ADAPT-ITT framework to adapt a Lifestyle Redesign^®^ occupational therapy intervention for diabetic foot ulcer self-management

**DOI:** 10.1093/tbm/ibag030

**Published:** 2026-06-02

**Authors:** Stacey L Schepens Niemiec, Lauren Styer, Jasmine Chapman, Elliot Myong, Ryan Moshtael, Christopher Lopez, Jocelyn Arteaga, Elaine Wong, Diana Motta Morales, Neel Kumar, Tze-Woei Tan

**Affiliations:** Mrs. T.H. Chan Division of Occupational Science and Occupational Therapy, University of Southern California, Los Angeles, CA, United States of America; Mrs. T.H. Chan Division of Occupational Science and Occupational Therapy, University of Southern California, Los Angeles, CA, United States of America; Mrs. T.H. Chan Division of Occupational Science and Occupational Therapy, University of Southern California, Los Angeles, CA, United States of America; Division of Vascular Surgery and Endovascular Therapy, Keck School of Medicine of USC, University of Southern California, Los Angeles, CA, United States of America; Mrs. T.H. Chan Division of Occupational Science and Occupational Therapy, University of Southern California, Los Angeles, CA, United States of America; Mrs. T.H. Chan Division of Occupational Science and Occupational Therapy, University of Southern California, Los Angeles, CA, United States of America; Mrs. T.H. Chan Division of Occupational Science and Occupational Therapy, University of Southern California, Los Angeles, CA, United States of America; Division of Vascular Surgery and Endovascular Therapy, Keck School of Medicine of USC, University of Southern California, Los Angeles, CA, United States of America; Division of Vascular Surgery and Endovascular Therapy, Keck School of Medicine of USC, University of Southern California, Los Angeles, CA, United States of America; Mrs. T.H. Chan Division of Occupational Science and Occupational Therapy, University of Southern California, Los Angeles, CA, United States of America; Division of Vascular Surgery and Endovascular Therapy, Keck School of Medicine of USC, University of Southern California, Los Angeles, CA, United States of America

**Keywords:** intervention adaptation, chronic disease management, stakeholder engagement, behavior change, rehabilitation, health promotion

## Abstract

**Background:**

Diabetic foot ulcers (DFUs) affect approximately 25% of people with diabetes and are associated with decreased quality of life, high healthcare costs, recurrent wounds, and amputation risk. Standard DFU care emphasizes offloading to promote healing; however, adherence is often poor due to behavioral, psychosocial, and everyday life challenges. These gaps highlight the need for person-centered interventions that support DFU self-management within daily routines.

**Purpose:**

To systematically adapt a Lifestyle Redesign^®^ occupational therapy intervention (LR-OT) for DFU self-management using the ADAPT-ITT framework.

**Methods:**

An iterative, stakeholder-engaged adaptation process was conducted across the first seven ADAPT-ITT phases: **A**ssessment, **D**ecision, **A**dministration, **P**roduction, **T**opical Experts, **I**ntegration, and **T**raining. Activities included targeted literature synthesis, theater testing with individuals with DFUs and DFU care providers, iterative material development, and expert consultation. Adaptation efforts focused on refining intervention content, delivery, and training supports to DFU-specific care demands while retaining alignment with LR-OT core elements.

**Results:**

The ADAPT-ITT process yielded an interventionist-ready, DFU-specific LR-OT package, including patient-facing materials, an interventionist resource guide, and a conceptual mapping linking LR-OT theoretical elements to DFU-targeted management activities. Stakeholder input informed coordinated refinements to content, session structure, and delivery to support feasibility within DFU care contexts.

**Conclusion:**

Application of the ADAPT-ITT framework supported a transparent, stakeholder-informed, and reproducible process for adapting an evidence-based occupational therapy intervention for a high-risk and medically complex population. This work illustrates how lifestyle-focused interventions can be systematically adapted for specialty care contexts, with subsequent testing needed to evaluate fidelity and effectiveness.

**Clinical Trial Information:**

#NCT06278935.

## Teaser

This study describes a structured process for adapting a lifestyle-focused occupational therapy intervention to support daily self-management for people with diabetic foot ulcers.

## Graphical abstract

**Graphical Abstract:**
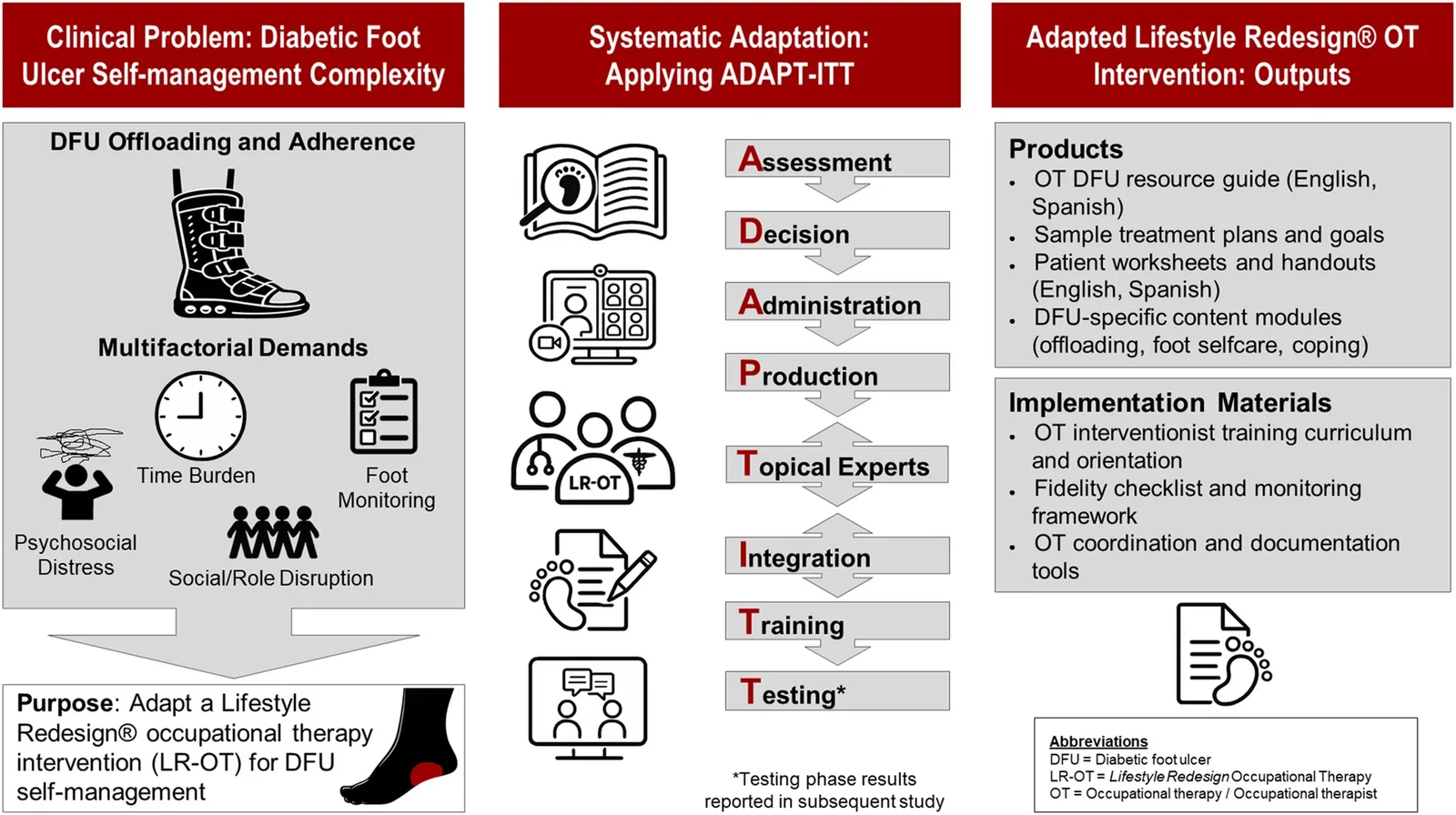

Implications

- **Practice: **Findings illustrate how a structured, iterative adaptation process—grounded in established core therapeutic elements and informed by patient and clinician perspectives—can help practitioners tailor existing interventions to the practical realities and contextual demands of specialized clinical populations such as those with diabetic foot ulcers.
- **Policy:** The transparent, stakeholder-engaged adaptation process used in this study highlights how policy structures that support collaborative, cross-disciplinary intervention development can strengthen the relevance and feasibility of programs intended for medically complex populations.
- **Research:** This study demonstrates that the ADAPT-ITT framework can be applied to medically complex specialty-care contexts, offering researchers a replicable model for systematically adapting behavioral and lifestyle interventions while maintaining alignment with core therapeutic principles.

## Introduction

Diabetic foot ulcers (DFUs) are full-thickness ulcerations below the ankle [1] that affect approximately 25% of individuals with diabetes over their lifetime [2]. DFUs represent a major public health burden, contributing to an estimated nine billion dollars in annual U.S. healthcare costs and accounting for 80% of diabetes-related amputations among the 1.6 million people affected [3]. DFUs often require prolonged periods to heal and ulcer recurrence is common; approximately 60% of individuals develop another DFU within three years of healing [4].

After addressing infection and ischemia, the mainstay of DFU management is pressure reduction through offloading to promote wound healing and prevent progression to limb-threatening complications. Offloading is achieved via removable devices (e.g. Controlled Ankle Motion [CAM] boot) or non-removable options (e.g. total contact cast). Non-removable devices are approximately 24% more effective at promoting wound healing [5], yet they are often declined due to mobility restrictions and discomfort. Although removable devices offer greater flexibility, adherence to prescribed offloading (i.e. consistent use of prescribed pressure-relieving devices or strategies during weight-bearing activities) remains suboptimal [6, 7]. For instance, patients with DFUs wore removable cast walkers during only 28% of their daily steps, underscoring the gap between prescribed offloading and real-world use [7].

Despite advances in wound care and offloading technology, contemporary DFU care models largely prioritize biomedical management [6, 8] and give limited attention to the psychosocial, behavioral, and everyday life factors that shape offloading adherence, wound care, and long-term self-management [9]. Functional limitations, competing self-care demands, disrupted routines, and treatment burden interact with diabetes distress and depressive symptoms, complicating sustained engagement in preventive and self-care behaviors [10]. Psychosocial distress related to uncertainty, risk of complications, and the possibility of amputation further exacerbates these challenges [11, 12]. Collectively, these factors highlight the need for interventions that address DFU self-management within the context of patients’ daily lives.

DFU care represents a clinical context in which intervention adaptation may be particularly critical. (In this manuscript, we use *adaptation* to refer to the planned, systematic process of modifying an EBI to improve its fit for the DFU care context, consistent with the ADAPT-ITT framework. Within this broader process, we use *tailoring* to denote fidelity-consistent, population-specific refinements to intervention content or delivery that preserve core LR-OT principles while aligning materials with DFU-specific clinical and contextual demands, consistent with the Framework for Reporting Adaptations and Modifications–Expanded (FRAME) [13].) Its medically intensive nature, prolonged care trajectories, and substantial behavioral demands related to DFU-specific offloading adherence and wound care place a significant burden on patients working to promote healing and prevent progression to limb loss. Existing evidence-based interventions (EBIs) developed under controlled conditions may not perform as intended when implemented in new contexts or populations without consideration for factors such as local workflows, resources, or lived experiences [14, 15]. Improving the fit between EBIs and real-world contexts through systematic adaptation has therefore become a central focus of implementation science. Such adaptations may occur at multiple levels, including changes to intervention content, delivery processes, or implementation context, while preserving core intervention elements, as described in the implementation science literature [13, 16]. Adaptation frameworks provide structured, theory-informed approaches to guiding these changes, supporting transparency, fidelity, and reproducibility across settings. Multiple frameworks have been described, including ADAPT-ITT and related models [16].

In DFU care, the need for contextual adaptation raises questions about which EBIs are best suited to address everyday self-management demands. Because DFU self-management is enacted through daily activities and routines, interventions that explicitly target integration of medically prescribed behaviors into everyday life warrant consideration. Occupational therapy (OT) represents one class of intervention with potential relevance for DFU self-management, particularly when delivered through theory-driven intervention models. To-date, OT contributions to DFU care have primarily taken the form of education- and task-focused interventions aimed at improving foot care behaviors and functional performance [17].

In this study, the ADAPT-ITT framework was selected to guide systematic assessment, selection, and adaptation of an EBI for DFU self-management. Originally developed to adapt HIV prevention interventions [18], ADAPT-ITT has since been applied across diverse health contexts and populations [19–21]. The framework comprises eight phases—**A**ssessment, **D**ecision, **A**dministration, **P**roduction, **T**opical Experts, **I**ntegration, **T**raining, and **T**esting—providing a systematic and transparent roadmap for adaptation. This paper reports the first seven phases of ADAPT-ITT, culminating in an interventionist-ready version of an adapted OT intervention for DFU self-management (with findings from the testing phase to be reported separately).

## Methods and results by ADAPT-ITT phase

This study was conducted through an academic-clinical partnership, with a research team co-led by an OT scientist who has certification in Lifestyle Medicine and Lifestyle Redesign^®^ and affiliation with an academic OT faculty practice, and a vascular surgeon with expertise in limb preservation and clinical appointments at two academic medical centers. Activities across the first seven ADAPT-ITT phases occurred between February 2024 and January 2025.

Table 1 provides an overview of the eight ADAPT-ITT phases, corresponding data collection activities, and key outputs generated during the adaptation process. An iterative, stakeholder-engaged approach to intervention adaptation was adopted, incorporating literature synthesis, theater testing sessions (small focus group, dyadic, and triadic interview formats), and clinician and expert consultation. Stakeholders involved across phases included occupational therapists, interdisciplinary DFU care providers, topical experts in limb preservation and diabetes care, and individuals with a history of DFUs. All procedures were approved by the University of Southern California Institutional Review Board under an expedited review with a determination of minimal risk. Human subjects research was conducted only during Phase 3 (Administration) and Phase 8 (Testing); other phases involved consultative program-development activities and did not constitute human subjects research. Informed consent was obtained from all Phase 3 and Phase 8 participants prior to study engagement.

**Table 1:** Overview of ADAPT-ITT phases, methods, and key outputs in the development of the Lifestyle Redesign^®^ diabetic foot ulcer management intervention.

ADAPT-ITT phase and purpose	Methods used	Outputs/key results
**Assessment: Gathering population needs and multilevel priorities.**	Interdisciplinary study team (i.e. vascular surgeon and *Lifestyle Redesign*-certified occupational therapist) conducted targeted literature synthesis identifying factors impacting diabetic foot ulcers (DFUs) 3 consultative meetings held with occupational therapists who work with at-risk populations at a large rehabilitation center to further identify needs and priorities	Assessment identified key lifestyle, behavioral, psychosocial, and service-related challenges affecting DFU self-management and care: Difficulty adhering to offloading regimens Limited integration of foot care routines into daily life Low self-efficacy for prevention behaviors Psychosocial distress related to activity restriction Limited physician referral to occupational therapy (OT) for DFU-specific self-management support Limited resources for Spanish-speaking patients, despite high DFU prevalence in Latinos
**Decision: Evaluating candidate evidence-based interventions and determining need for adaptation.**	Regular study team meetings, leading to consensus that the *Lifestyle Redesign* occupational therapy (LR-OT) approach would be appropriate if adapted to meet the needs and priorities of the DFU population	The study team identified key areas requiring adaptation to tailor LR-OT for DFU care, including: Integrating DFU-specific content (e.g. offloading, wound care routines, footwear management) Reinforcing coordination with DFU medical providers Aligning intervention goals with wound-healing priorities
**Administration: Pretesting the intervention prototype with a representative sample.**	Theater testing conducted via Zoom videoconference with 5 sessions of key stakeholders (*N *= 14; small-group, triadic, and dyadic formats) 2 brief video clips shown of simulated LR-OT treatment sessions with a patient with diabetes (no specific DFU content) Semi-structured interview guide used to elicit feedback on the LR-OT approach	Priorities identified from theater testing discussions included: LR-OT topical areas and strategies viewed as directly applicable to DFU self-management Components and content to be retained but modified for DFU-specific application (e.g. adapting physical activity and self-care routines to offloading requirements; reinforcing health self-advocacy and patient–DFU provider communication) Content that could be backgrounded or less of a focus (e.g. detailed dietary behavior content for diabetes control) Implementation considerations (e.g. earlier intervention in DFU care trajectory, flexible scheduling and session duration, hybrid delivery, interdisciplinary coordination)
**Production: Developing and refining the intervention to meet population needs.**	Iterative development by interdisciplinary study team via internal team meetings to discuss intervention structure Written and oral feedback circulated among team members to draft and refine intervention content, resources, and training materials Spanish translation of English materials	Initial working draft of *Lifestyle OT Intervention for People Managing Diabetic Foot Ulcers and Offloading: An Occupational Therapist Resource Guide* Initial working drafts of patient-facing worksheets and materials in English and Spanish
**Topical Experts: Engaging topical experts to review and refine the intervention.**	18 long-form, individual consultations conducted with experts from 5 disciplines with in-depth knowledge of DFU care and/or diabetes lifestyle intervention: podiatric surgery, physical therapy, OT, vascular nursing, and prosthetics/orthotics	Priorities for refinement identified: Addressing psychosocial barriers to offloading and foot self-care Emphasizing integration of foot self-care routines Necessary patient advocacy for optimal DFU care Educating and reinforcing information on offloading device use and compliance
**Integration: Synthesizing feedback from all previous stages to refine the intervention.**	Systematic incorporation of feedback from topical experts, individuals with DFU experience, and DFU care team members Cross-walking stakeholder input with LR-OT core elements to resolve inconsistencies and enhance clinical relevance Team-based review and iterative refinement to ensure coherence across all materials and alignment with DFU care workflows	Finalized version of the interventionist resource guide Finalized patient-facing worksheets and materials in English and Spanish, incorporating DFU-specific content Preliminary conceptual map linking LR-OT elements to DFU-targeted activities
**Training: Preparing personnel to deliver the intervention with fidelity.**	Conducted 3-hour virtual orientation led by OT co-principal investigator, covering study aims, intervention framework, DFU-specific considerations, and treatment session flow Provided 2-week independent self-study period using interventionist resource guide (readings, sample treatment plans, DFU-specific patient materials) Offered ongoing consultation with co-principal investigators during training Second LR-OT interventionist shadowed the primary LR-OT for one week to observe intervention delivery Developed and implemented a structured fidelity monitoring procedure using a checklist created in the Production phase Integrated fidelity monitoring procedures into interventionist training and supervision	Trained OT interventionists prepared to deliver the adapted DFU-specific LR-OT intervention Standardized training materials and fidelity monitoring procedures established to support consistent delivery Implementation-ready fidelity monitoring framework in place for subsequent testing
**Testing: Testing the intervention with patients and gathering outcomes.**	2 trained LR-OTs delivered the adapted intervention to patients with active DFUs in a pre-post single arm pilot study Data collection included participant feedback, clinician feedback, and pre–post patient assessments of functional performance, diabetes distress, satisfaction with DFU care, offloading knowledge and attitudes, and wound status (electronic medical record chart review)	Feasibility, acceptability, and preliminary patient outcome data generated Detailed pilot results to be reported in a separate manuscript

### Phase 1: Assessment

#### Methods

The *Assessment* phase involved identifying population needs and multilevel factors affecting DFU self-management to inform subsequent intervention selection and adaptation. A targeted literature synthesis examined DFU prevention and management, offloading adherence, psychosocial and behavioral determinants of wound healing, and chronic disease self-management interventions relevant to DFUs. The scope and interpretation of the literature synthesis were informed by the vascular surgeon co-principal investigator’s clinical experience and prior needs assessment work, including patient-reported findings on the physical, psychosocial, and financial impacts of DFU and amputation [11] and a recent comprehensive clinical review of DFU care [8]. Interpretation was further shaped by the anticipated implementation context within a Los Angeles County public health–academic OT partnership.

Given DFU self-management requires patients to integrate medically prescribed behaviors (e.g. offloading, wound care, foot inspection) into everyday activities and routines, the Assessment phase examined intervention domains defined by their primary focus on supporting daily activity performance and long-term self-management. OT represents one such domain due to its focus on enabling participation in daily life and supporting chronic disease self-management through activity-based strategies. Accordingly, OT-based interventions were explored alongside other behavioral and educational approaches without presupposing their suitability for adaptation.

To complement the literature synthesis, three consultative meetings were held with occupational therapists at a local Los Angeles rehabilitation hospital serving a diverse, safety-net patient population, many with diabetes and foot complications. During these meetings, the research team summarized assessment findings, and the occupational therapists reflected on DFU-related challenges observed in their practice and identified gaps in existing DFU education and support resources from an occupational perspective.

#### Results/outputs

Assessment activities identified interconnected lifestyle, behavioral, psychosocial, and service-related challenges influencing DFU self-management and care. At the patient level, challenges included difficulty incorporating offloading and foot care into daily routines and low self-efficacy to sustain preventive behaviors. At the service and system level, apparent issues were the absence of physician referrals for DFU-specific OT services and limited availability of linguistically appropriate resources for Spanish-speaking individuals—a group disproportionately impacted by DFUs in the regional context.

### Phase 2: Decision

#### Methods

For the *Decision* phase, findings from the Assessment phase, including the literature synthesis, clinical context, and OT consultations, were integrated to evaluate candidate EBIs and determine suitability for adaptation to DFU care.

#### Results/outputs

Candidate interventions were evaluated according to pragmatic criteria: theoretical grounding in daily routines and habit formation, capacity to address multilevel behavioral determinants, prior evidence of feasibility or effectiveness in chronic conditions, and feasibility of delivery within clinical workflows. Current DFU interventions emphasize biomedical management (i.e. wound care, infection treatment, glycemic control) as primary drivers of healing, with comparatively little attention to behavioral integration, daily routines, and long-term self-management [6, 8]. OT interventions, particularly education-focused foot care programs, have demonstrated effectiveness in improving diabetic foot self-care behaviors, including integration of foot inspection and hygiene into daily activities [17]. However, these were not theory-driven EBIs and appeared limited to discrete education and skill acquisition, e.g. [22] without attending in depth to the full complexities of integrating DFU self-management habits and routines long-term.

Among the interventions reviewed, *Lifestyle Redesign* OT (LR-OT) emerged as a theory-driven EBI delivered within OT practice and guided by the *Lifestyle Redesign* framework. In this context, the framework provides the theoretical and methodological foundation, while LR-OT is the operationalized OT intervention shaped by the framework [23, 24]. LR-OT is defined by core elements that include: (i) *core characteristics* (i.e. focusing on daily activity orchestration, applicability to lifestyle challenges, collaborative therapist-client process with individualization, health-relevant outcomes, and delivery by trained OT practitioners) that distinguish LR-OT from other approaches; (ii) *core domains* (i.e. meaningful occupations, habits, and routines; chronic condition management; psychological well-being; environment and context; and advocacy and healthcare access) that guide intervention content; and (iii) *core techniques* (i.e. patient education, occupational self-analysis, narrative reasoning, problem solving, and autonomy-enhancing communication) that shape therapeutic processes [23, 24]. These elements align conceptually and practically with the multilevel, routine-dependent DFU challenges identified in Phase 1. On that basis, LR-OT was selected for systematic, context-based adaptation to address DFU self-management across the DFU continuum (active wound management to recurrence prevention to limb preservation).

When operationalized for specific populations or contexts, LR-OT has demonstrated feasibility and benefits for function, self-management, and quality of life [23], including for safety-net patients with diabetes [25]. DFU self-management, however, presents distinct demands relative to prior applications of LR-OT. DFU care requires sustained adherence to risk-sensitive behaviors alongside episodic acute medical events (e.g. infection, urgent surgical intervention) that can disrupt routines [26–28]. Moreover, DFU care is delivered within specialized, limb-preservation teams where OT is not routinely embedded. Accordingly, the research team decided to adapt versus adopt LR-OT, to facilitate integration of DFU-specific content (e.g. offloading routines, wound surveillance, footwear management), reinforce coordination with the interdisciplinary DFU care team, and align intervention goals both with patient-centered needs and wound-healing priorities.

### Phase 3: Administration

#### Methods

The *Administration* phase involved pre-testing an intervention prototype with intended users to inform adaptation prior to implementation. The research team used theater testing, a structured formative method in which participants observe simulated intervention sessions prior to adaptation, and provide feedback on relevance, format, and acceptability.

A total of five theater testing sessions were conducted (*N *= 14), using small-group qualitative formats, including one small focus group (*n *= 4), two triadic interviews (*n *= 3 each), and two dyadic interviews (*n *= 2 each). This phase of the study was initially designed to include three small focus groups of approximately five participants each to support manageable yet interactive discussion. However, to accommodate scheduling constraints among interdisciplinary providers while preserving participation, session formats were flexibly adapted to ensure representation across clinical roles, consistent with adaptive qualitative designs in applied settings [29]. Participants included individuals with a history of DFUs and members of interdisciplinary DFU care teams (podiatry, vascular surgery, orthotics/prosthetics, nursing, and medicine; see Supplementary Table S1). Participants were recruited through the research team’s clinical and professional networks via word-of-mouth. Sessions lasted about 90 min.

The use of small-group, dyadic, and triadic formats is consistent with contemporary qualitative research practices that prioritize interactive exchange and depth of discussion, particularly among participants with specialized or complementary roles [30–32]. This approach enabled inclusion of diverse perspectives patient and provider perspectives while maintaining the interactive features central to theater testing.

All sessions followed the same semi-structured guide and theater testing procedures. Participants viewed two brief videos (6–14 min each) depicting simulated non-DFU-specific, telehealth LR-OT sessions for adults with diabetes (Supplementary Table S1). Videos presented diabetes-relevant lifestyle topics (i.e. physical activity and energy management, mental health and well-being, foot self-care, preparing for healthcare visits, diet and nutrition) and illustrated LR-OT core principles [23, 24]. Video combinations varied across sessions to ensure coverage of key topics within the available time.

Sessions were facilitated by study investigators with support from trained research staff using the interview guide to systematically elicit feedback on relevance, format, and acceptability. Consistent with ADAPT-ITT theater testing procedures, structured, domain-focused prompts were used to assess participant reactions to intervention content, delivery, and materials and to identify needed adaptations. Although the original framework describes the use of brief surveys to initiate discussion [18], structured qualitative discussion was used to capture detailed, context-specific feedback for a flexible, non-modular intervention, as reflected in recent ADAPT-ITT applications, e.g. [33]. Relevance was examined through participant perspectives on applicability to DFU self-management; format via feedback on delivery mode, session structure, and alignment with existing DFU care workflows; and acceptability was evaluated through discussion of perceived appropriateness and patient willingness to engage.

Recordings were auto-transcribed verbatim and imported to Dedoose qualitative software (SocioCultural Research Consultants, LLC, Los Angeles, CA) for analysis. Two trained research personnel independently reviewed the transcripts and coded the data using a directed, question-guided approach informed by the semi-structured interview guide and adaptation-relevant categories (e.g. content, delivery, contextual relevance), consistent with directed content analysis [34]. Coded documents were compared and reconciled through team discussion to reach consensus. This pragmatic analytic approach supported formative identification of adaptation needs rather than theory generation.

#### Results/outputs

Theater testing sessions yielded feedback across four thematic areas that informed subsequent adaptation decisions: (i) LR-OT topical categories viewed as essential to retain, (ii) content requiring tailoring for DFU-specific needs, (iii) topics viewed as lower priority for DFU management, and (iv) practical implementation considerations (see Table 2).

**Table 2:** Stakeholder perspectives on content retention, adaptation, and implementation considerations for a diabetic foot ulcer (DFU)-specific Lifestyle Redesign^®^ occupational therapy (LR-OT) intervention.

Thematic category	Interpretation/analytic summary	Illustrative quotes (participant type)	Adaptation implications/recommendations
**LR-OT categories of topics to retain**	Participants emphasized the importance of retaining key categories of occupations/topics addressed in the original intervention framework, including emphases on mental and emotional well-being, social support as a part of a patient’s context, and engagement in and attention to meaningful occupations and routines as foundational for sustaining motivation and identity during DFU management.	“I think mental health is a big piece of not only offloading in your feet, but also diabetes in general.” (DFU patient) “It’s assuming that the patient has family and friends that’ll help them […] but it’s not like that many times.” (Provider) “[Using a knee scooter] gave me that sense of independence […] even if it was something as simple as going into the kitchen to pick up my plate for dinner.” (DFU patient) “Sometimes, […]there’s so much going on in life other than your diabetes and your health that you tend to, at times, forget what you should be doing.” (DFU patient)	Retain focus on mental and emotional well-being as a key intervention component Explicitly address variability in social support that is critical to successful DFU self-management Maintain emphasis on meaningful routines and identity preservation during offloading
**Content to tailor for DFU-specific needs**	Participants identified the need to adapt intervention content from general diabetes management to address DFU-specific physical constraints and contextual challenges, such as offloading-compliant daily activity, considerations of pain or sensation changes, adaptive equipment use, and structured foot- and wound-care routines.	“I bought a scooter just to take my dog for a walk […] it helps me prepare my day because if he could still go, then I could still go.” (DFU patient) “If you have an injury, you’ll probably modify the activity because it causes pain or discomfort. But in this case, that’s less likely to happen, since there’s no pain […] it’s more of an issue with a lack of sensation.” (Provider) “Things that you need to remember for your wound […] just having a personal checklist […] it’ll kind of help organize thoughts.” (DFU patient)	Adapt physical activity content to emphasize offloading-safe participation Integrate guidance on adaptive equipment and mobility aids to support safe engagement in meaningful occupations Develop structured tools (e.g. checklists) to support daily footcare, wound care, and DFU self-monitoring
**Topics viewed as lower priority**	Providers and patients viewed diet-related content, which is often a focus for general diabetes management, as less central or high priority for DFU-specific self-management among patients with longstanding diabetes, though still relevant to overall disease control.	“For somebody who just got a diabetic foot ulcer, they should pretty much already have a handle on the eating aspects of the disease.” (DFU patient) “This [management of an irremovable offloading device] would require a completely different session from controlling and treating diabetes and how to keep your feet clean and a diet session.” (Provider)	De-emphasize general diet education to allow time to discuss more DFU-focused content Integrate dietary discussions selectively or via referral to a nutritionist when needed
**Practical implementation considerations**	Participants highlighted the importance of early intervention, flexible scheduling, hybrid delivery formats, and strong interdisciplinary coordination with a large DFU care team to support feasibility and uptake within DFU care settings.	“The importance of doing everything on the checklist doesn’t hit anyone till it’s too late.” (DFU patient) “Especially at the beginning, more frequent visits are important and then they can be spaced out once things are starting to improve or show some healing potential.” (Provider) “I’ve never had a tele-visit, but I can see where that would be very beneficial. Maybe somebody is not too mobile, but one-to-one. I would be open to whatever in my case, […] I just want to get healed.” (DFU patient) “As an orthotist, if I had a summary report […] that would be awesome because […] I’d have a snapshot of their lifestyle even better than I can interview a patient.” (Provider)	Introduce intervention early in the DFU care trajectory and tailor to patient needs as DFU wound healing unfolds Offer flexible scheduling and hybrid (in-person/telehealth) delivery options to improve access and uphold patient-centeredness Strengthen coordination and information sharing across DFU care teams and refer to members of the team when relevant

Participant identifiers reflect role (DFU patient or provider). Quotes are illustrative and anonymized.

##### LR-OT topical categories to retain

Participants emphasized the enduring relevance of several categories of topics/occupations addressed in general diabetes LR-OT, particularly attention to mental and emotional well-being, social support for chronic condition management, and engagement in meaningful occupations and routines. These topics were viewed as central to sustaining motivation, identity, and adherence during prolonged offloading and DFU recovery.

##### Content to tailor for DFU-specific needs

Participants identified multiple opportunities to tailor existing LR-OT intervention content available for general diabetes management to address DFU-specific demands. Recommendations focused on supporting offloading-safe participation in daily activities and accounting for altered pain and sensation that come with active wounds. Participants also noted the need to incorporate guidance on adaptive equipment, protective footwear, and mobility aids. Building or strengthening structured routines for daily foot and wound care and self-monitoring was also viewed as important.

##### Elements viewed as lower priority

Diet-related content was the primary topic identified by participants as lower priority for DFU-focused intervention sessions. Patients expressed that for those with longstanding diabetes, dietary education was often already familiar and less immediately relevant to the challenges of offloading and wound management. Participants acknowledged that, while diet remains important for overall diabetes control, DFU-specific sessions should prioritize content directly related to offloading management and foot selfcare.

##### Practical implementation considerations

Participants highlighted several practical considerations affecting feasibility and uptake. These included introducing LR-OT early in the DFU care trajectory and offering flexible scheduling and hybrid delivery formats. Stronger interdisciplinary coordination and information sharing across DFU care providers was also emphasized. Collectively, these findings informed subsequent topical expert consultation and integration phases.

### Phase 4: Production

#### Methods

The *Production* phase focused on iterative development and refinement of draft intervention materials and implementation tools to operationalize LR-OT for DFU self-management and occurred in parallel with topical expert consultations (Phase 5). The process was intentionally iterative and non-linear, with materials refined through successive cycles of review, feedback, and revision. The team reviewed existing LR-OT resources used in diabetes self-management and general health promotion to identify adaptable content. Draft DFU-specific materials were developed following theater testing and iteratively refined as additional insights were gathered from topical experts and preliminary, formative feedback from OT interventionists during early implementation activities.

As draft intervention components evolved, the OT co-principal investigator also led development of a fidelity monitoring checklist to support quality assurance during implementation (see Supplementary Table S2). Checklist content was informed by LR-OT literature, certification training materials, and professional expertise. It was designed to capture LR-OT core characteristics, domains, and techniques, along with DFU-specific self-management alignment. The checklist was informally piloted on early treatment sessions to assess clarity and usability, and minor revisions were made prior to use in training and implementation.

#### Results/outputs

Production outputs included initial drafts of patient-facing handouts and activity guides, along with preliminary materials for the occupational therapist training package (e.g. interventionist resource guide). Together, these products formed a cohesive and adaptable intervention package grounded in LR-OT core elements and tailored to DFU care challenges (see Table 3). Materials emphasized adherence to offloading regimens, wound monitoring, and psychosocial coping while maintaining focus on lifestyle balance and self-management. Iterative revisions improved readability, relevance, and clinical usability. A structured fidelity checklist was produced to support implementation interventionist training.

**Table 3:** Examples of DFU-specific activities and strategies developed through ADAPT-ITT Production and Integration phases and aligned with Lifestyle Redesign^®^ core domains and characteristics.

LR-OT core characteristic or domain	Representative Diabetic Foot Ulcer (DFU) challenges	Sample DFU-specific activities/strategies
**Focus on orchestrating daily activities/lifestyle patterns/routines**	Integrating daily foot-care tasks into established routines Adjusting occupations and mobility patterns to maintain foot protection Balancing offloading requirements with valued activities and responsibilities	Guided analysis of routines to identify opportunities to embed daily foot checks and necessary wound care activities Collaborative modification of daily activities to reduce harmful weight-bearing exposure Development of individualized physical activity plans aligned with offloading guidelines
**Applicable to lifestyle-related challenges**	Chronic pain and discomfort that disrupt daily routines Reduced mobility and activity limitations related to offloading Cognitive load required to remember, plan, and carry out essential DFU care behaviors	Guided exploration of how pain, mobility restrictions, and foot self-care demands affect daily occupations, such as physical activity and household routines Practice with strategies for managing stress, supporting physical activity, and sustaining valued occupations despite limitations Problem-solving approaches to mitigate social participation barriers (e.g. finding alternative ways to stay socially connected while adhering to offloading guidelines)
**Therapist-client collaboration evident**	Difficulty adhering to complex treatment plans (e.g. offloading, wound care, activity modifications) Challenges adopting and sustaining behavioral changes over time Need for support in making autonomous, informed decisions about DFU care routines	Collaborative development of strategies related to DFU care, pain management, mental well-being, and activity planning, emphasizing participant autonomy and lived expertise Use of reflective dialogue and shared decision-making (e.g. offering choices about session focus, co-creating plans such as for safe physical activity) Guided self-reflection through open-ended questions to support individualized goal setting, problem-solving, and follow-through with DFU care plan
**Aimed at improving health and well-being outcomes**	Compromised physical and emotional well-being due to pain, wound severity, mobility limits, and treatment burden Difficulty maintaining behaviors that support wound healing, diabetes control, and overall health Stress, poor sleep, and fluctuating blood glucose that undermine day-to-day functioning and recovery	Supporting diabetes self-management, safe physical activity, and routines that promote wound healing and preserve mobility Incorporating stress-regulation strategies to enhance sleep, emotional balance, and day-to-day stability, linking these practices to long-term DFU outcomes Monitoring progress using participant feedback and functional observations across domains such as daily routines, wound-protective behaviors, and self-efficacy in managing health and lifestyle demands
**Delivered by an LR-OT trained practitioner**	Limited access to providers trained in behavioral and lifestyle approaches tailored to DFU self-management Difficulty integrating DFU-specific offloading, wound care, and activity modifications across multiple providers and care settings Challenges coordinating recommendations from multidisciplinary teams in ways that fit patients’ daily lives	Intervention delivered by 2 licensed OTs with advanced clinical credentials trained in LR-OT and specialized expertise relevant to diabetes management Use of OT-guided collaborative problem-solving to help patients integrate guidance from podiatry, wound care, endocrinology, and others into coherent daily routines Tailored educational and planning tools created by LR-OTs to translate multidisciplinary DFU recommendations into actionable, sustainable daily habits
**Meaningful occupations, habits, routines**	Disruption of daily living tasks due to mobility limitations, pain, and offloading requirements Social isolation stemming from restricted mobility or fear of infection Difficulty integrating DFU management tasks into valued routines	Facilitated reflection on daily habits and routines to support safe, meaningful engagement in physical activity while maintaining adherence to offloading recommendations Collaborative development of structured self-care strategies, such as implementing a daily foot-check routine and using wound monitoring tools Co-organization of routines to integrate DFU care into valued daily occupations and to promote well-being Co-creation of realistic, health-promoting activity goals aligned with rehabilitation plans and lifestyle priorities
**Occupations relevant to wellness and/or chronic condition management**	Difficulty recognizing and responding to early signs of diabetic foot complications Challenges maintaining routines that support overall health and DFU self-management Reduced physical activity due to pain, offloading, or mobility limitations	Training and support to recognize and respond to early signs of diabetic foot complications, emphasizing timely action and integration into daily self-management Guided foot self-care education and structured daily foot-check routines, exploring connections between sleep, stress, blood glucose, and wound healing Integration of offloading and circulation-promoting strategies into daily routines (e.g. seated stretching, activity pacing, strength exercises, adaptive tools use) Organization of daily occupations to support energy conservation, healthy routines, and stress management, promoting consistent DFU self-care and overall well-being
**Mental health and psychological well-being**	Anxiety and depression related to wound healing and treatment burden Frustration or discouragement due to prolonged recovery Stress and emotional strain impacting adherence to DFU self-management routines	Support for recognizing and managing emotional responses (e.g. anxiety, helplessness, information overload) through guided reflection and self-regulation strategies Development of coping skills, self-efficacy, and confidence to independently manage daily routines and DFU care Collaborative problem-solving to address psychosocial barriers to DFU self-management and promote engagement in meaningful daily activities
**Environment and context**	Need for home environment modifications to support safe foot self-care and mobility Need for workplace modifications to accommodate offloading and activity limitations Environmental barriers that affect adherence to DFU care routines and overall participation	Development of fall-prevention strategies through environmental analysis and modification, such as optimizing foot-check locations, sleeping/bathing areas, and safe movement within offloading restrictions Support for incorporating DFU self-management into broader home and healthcare contexts, such as troubleshooting access to adaptive tools and integrating cues like reminder alarms Exploration of community and life-space mobility, identifying environmental stressors and supports that influence safety, offloading adherence, and engagement in meaningful activities
**Healthcare access and advocacy**	Difficulty accessing specialized care for DFU management Challenges with health literacy, understanding medical instructions, or navigating care Insurance, financial, or logistical barriers impacting adherence to treatment	Enacting time management strategies to coordinate multiple healthcare appointments and troubleshoot access to adaptive tools (e.g. long-handled mirrors, reachers) to support consistent DFU self-care within daily routines Developing preparation and engagement strategies for care appointments, addressing barriers such as insurance changes, care team transitions, and inter-provider communication Building communication and self-advocacy skills to strengthen interactions with family and healthcare providers, supporting offloading adherence and overall treatment engagement

Examples are illustrative and not exhaustive. Activities and strategies represent intervention-level adaptations rather than within-session individual tailoring. LR-OT core characteristics and domains denote established theoretical elements of the Lifestyle Redesign^®^ approach and are shown to reflect conceptual mapping of DFU-specific activities.

### Phase 5: Topical experts review

#### Methods

The *Topical Experts* phase engaged external professionals with specialized knowledge of DFU care or LR-OT to inform clinical fit, relevance, and feasibility. Consultation sessions consisted of individual, semi-structured discussions lasting approximately 45–90 min, and were conducted as draft materials were generated and revised during the Production phase. Five experts representing podiatric surgery, physical therapy, vascular nursing, prosthetics/orthotics, and OT (LR-OT-trained) were recruited through the study team’s professional networks. Across 18 consultations, experts reviewed evolving intervention content, materials, and procedures and provided practice-based feedback. Fit, relevance, and feasibility were assessed qualitatively through discussion of alignment with DFU clinical workflows, appropriateness for patient needs and capabilities, and practicality of delivery in real-world DFU care settings. Consultations were documented through detailed notes and transcript review. Feedback was synthesized and organized into topical categories relevant to intervention refinement, including content accuracy, clinical integration, patient burden, workflow fit, and OT role clarity. Recommendations were iteratively incorporated into revised materials and cross-checked with findings from earlier ADAPT-ITT phases and DFU best-practice guidelines.

#### Results/outputs

Topical experts endorsed LR-OT as a valuable behavioral intervention complement to standard DFU care, particularly for supporting patient self-management and interdisciplinary coordination. Feedback led to refinements emphasizing practical integration of offloading, wound care, and psychosocial support within patients’ daily routines. Several consultations highlighted the need for clear, actionable patient education tools, including wound monitoring checklists, foot hygiene and toenail care worksheets, and guidance for communicating effectively with DFU care teams. These tools were viewed as important for promoting independence among patients managing complex regimens with limited health literacy or support. Input across disciplines also underscored the importance of mobility and fall prevention during offloading. Experts noted tension between adhering to offloading prescriptions and maintaining daily activities or social participation. In response, experts recommended explicit inclusion of gait safety, physical activity planning, and strategies for balancing healing with meaningful engagement in life roles.

The consultations also emphasized the psychosocial and socioeconomic dimensions of DFU management, including anxiety about wound appearance, financial and insurance barriers, and limited social support. To better integrate OT within DFU clinical workflows, recommendations included development of shared documentation tools and resources to familiarize OT interventionists with key aspects of DFU management (e.g. types and functions of offloading devices). Together, this input informed refinements to intervention materials and structure. Recommendations from topical experts were interpreted and incorporated in a manner intended to adapt and augment DFU-specific content while preserving the established core elements of LR-OT, as described in the Decision phase and in further detail in Schepens Niemiec et al. (In Press) [24] and Pyatak et al. (2022) [23].

### Phase 6: Integration

#### Methods

The *Integration* phase focused on synthesizing materials and feedback generated across prior phases to produce a cohesive, finalized intervention package. Refinements were applied to patient-facing materials, OT interventionist resources, and training protocols to ensure consistency, clarity, and alignment with DFU-specific needs, LR-OT core elements [23, 24], and anticipated clinical workflows. Integration emphasized coherence across components rather than introduction of new intervention content.

A key integration activity involved development of a preliminary conceptual mapping linking LR-OT core characteristics and domains to specific DFU-targeted intervention activities (see Table 3). This mapping was developed through structured synthesis of findings from earlier phases and iterative review of finalized intervention materials to ensure conceptual alignment and fidelity to LR-OT theoretical mechanisms of action (Detailed specification of adaptation types, levels, rationales, and intended outcomes using the FRAME framework is reported elsewhere [24]; the present manuscript focuses on the ADAPT-ITT-guided process through which these elements were integrated into a unified intervention package.).

#### Results/outputs

The Integration phase resulted in a finalized intervention package, including patient-facing materials, OT intervener resources, and interventionist orientation protocols. Materials reflected prior-phase feedback and were aligned to address key DFU self-management challenges (e.g. offloading adherence, foot health monitoring, activity modification, social support integration), with guidance supporting flexible delivery for hybrid in-person and telehealth clinical contexts. The conceptual mapping connected LR-OT core characteristics and domains to specific DFU-targeted activities, providing a structured framework for consistent intervention delivery and fidelity monitoring. Finalized intervention components retained LR-OT core characteristics and domains while incorporating adaptations of existing LR-OT strategies and DFU-specific tools necessary to address wound care and offloading demands. Integration activities ensured the intervention was cohesive, operationally feasible, and prepared for pilot testing.

### Phase 7: Training

#### Methods

The *Training* phase focused on preparing occupational therapists with prior formal training in LR-OT to deliver the adapted DFU-specific intervention. Because interventionists were already formally trained in LR-OT through professional education and certification pathways, Phase 7 training emphasized orientation to DFU-specific adaptations rather than re-training in the original underlying LR-OT intervention.

The primary OT interventionist completed required human subjects protection training for research participation, followed by a three-hour virtual orientation led by the OT co-principal investigator. The orientation introduced study aims, reviewed the adapted intervention structure, and provided a guided walkthrough of an interventionist resource guide. The guide included DFU-specific clinical background, tailored application of LR-OT strategies, sample treatment plans and goals, patient-facing materials, and guidance on documentation and communication procedures. Following orientation, the OT interventionist completed a two-week period of independent review of the resource guide, with optional consultation available as needed. To support quality assurance during implementation, a structured fidelity monitoring procedure using a checklist developed during the Production phase was established. A second OT interventionist was later onboarded and received the same orientation and resource materials, followed by a one-week shadowing period with the original interventionist.

#### Results/outputs

Training outputs included completion of DFU-specific orientation for OT interventionists, dissemination of the finalized OT interventionist resource guide, and establishment of a standardized fidelity monitoring process to support consistent delivery. Together, these activities prepared OT interventionists to deliver the adapted DFU-specific LR-OT intervention in a manner intended to preserve alignment with established LR-OT principles.

### Phase 8: Testing

#### Methods

The adapted DFU-specific LR-OT intervention was pilot tested to examine feasibility and acceptability, and to generate preliminary patient outcomes data. Adults with DFUs were enrolled and received LR-OT services as part of this phase. Data collection included patient-reported measures, electronic medical record review, and implementation feedback to support further refinement and subsequent study design.

#### Results/outputs

Pilot testing was completed, resulting in generation of feasibility, acceptability, and preliminary patient outcomes data to inform subsequent analysis and study design. Detailed methods and findings from the Testing phase will be reported separately.

## Discussion

This study applied the ADAPT-ITT framework to guide systematic, context-based adaptation of an evidence-based OT intervention for adults with DFUs. By integrating behavioral, lifestyle, and medical perspectives, the adaptation aimed to address persistent gaps in DFU care related to offloading adherence, self-management demands, and psychosocial adjustment. Although advances in wound care and limb preservation have improved biomedical outcomes, current DFU care pathways rarely incorporate interventions targeting habits, routines, and psychosocial barriers that shape self-management [11, 27, 28]. Furthermore, occupational therapists are not typically included in multidisciplinary DFU care teams, although their expertise could substantially address these essential factors. Randomized trials have demonstrated the effectiveness of LR-OT for improving diabetes self-management and quality of life in primary care, underscoring the broader relevance of OT lifestyle-focused approaches within diabetes-related conditions [25].

Prior ADAPT-ITT studies have largely centered on community-based HIV or public health interventions, e.g. [35, 36]. By applying ADAPT-ITT within specialty DFU care, this study extends its use to contexts requiring integration of complex medical regimens, functional limitations, and daily routines.

Stakeholder engagement across ADAPT-ITT phases informed coordinated adaptations to intervention content, session structure, and delivery. Content adaptations emphasized DFU-specific self-management demands such as offloading adherence, wound monitoring, mobility safety, and integration of these tasks into daily routines. Session structure was refined to allow flexible pacing and topic emphasis based on wound status and competing care demands, while delivery adaptations supported hybrid telehealth models and coordination within interdisciplinary DFU care teams. These changes were integrated to strengthen contextual fit and feasibility while preserving alignment with LR-OT core elements. This approach aligns with prior research demonstrating that lifestyle interventions informed by stakeholder-engaged activities can yield meaningful improvements in health behaviors and physical and mental health outcomes in chronic disease contexts [37]. By systematically linking LR-OT core elements to DFU-specific needs, the adaptation also produced detailed intervention materials and conceptual mappings that support fidelity monitoring and training for OT interventionists, which represents a critical step for future scale-up [38].

Interdisciplinary collaboration was central to the adaptation process. OT expertise informed decisions about which lifestyle domains to emphasize, how DFU self-management tasks could be embedded into daily routines, and how intervention strategies could be operationalized within real-world care contexts. Collaboration with care providers and individuals with lived DFU experience added practice-based and patient-centered insights, illustrating how occupation-centric approaches can be integrated into specialty medical care.

Several limitations should be acknowledged. The adaptation process involved a relatively small number of patients, clinicians, and experts, many of whom were recruited through the research team’s networks, which may limit transferability. Although intervention materials were translated into Spanish, monolingual Spanish-speaking patients or providers were not included in the adaptation process. Patient perspectives were drawn from individuals willing to participate in qualitative study activities, potentially introducing selection bias. Additionally, while ADAPT-ITT provided structure, some phases were necessarily non-linear, which may complicate replicability in other contexts. As the adaptation relied partly on self-reported behaviors, offloading practices, and psychosocial needs, responses may have been subject to recall or social desirability bias. Variation in session format (small focus group, triadic, and dyadic interviews) may have influenced interaction dynamics within sessions; however, this approach enabled inclusion of diverse interdisciplinary perspectives that would likely not have been captured using a fixed group format. Finally, the current paper does not present feasibility or outcome data from the pilot testing phase; those results will be reported separately.

The extent of DFU-specific content integrated into the adapted intervention has implications for subsequent evaluation. Future studies should assess whether modifications enhance engagement, adherence, and self-management without increasing treatment burden. Evaluation designs should prioritize feasibility, acceptability, and fidelity across diverse clinical workflows. Attention to mechanisms of change, such as routine integration, self-efficacy, and care coordination, will be critical for understanding how lifestyle-focused interventions function within medically complex care contexts.

## Conclusion

Using the ADAPT-ITT framework provided a transparent, replicable process for adapting an evidence-based lifestyle intervention to a new clinical context and population. The resulting DFU-specific LR-OT intervention demonstrates how systematic, stakeholder-informed adaptation can be structured to maintain alignment with core therapeutic elements while improving contextual fit for medically complex care settings. This work highlights the potential role of OT in DFU management by targeting daily routines and everyday life challenges that are not typically addressed in standard limb preservation care pathways. These findings contribute to the growing literature on evidence-informed adaptation of behavioral interventions and offer a replicable model for integrating lifestyle-focused approaches within specialty medical care.

## Supplementary Material

ibag030_Supplementary_Data

## Data Availability

De-identified data from this study are not available in a public archive. De-identified data from this study will be made available (as allowable according to institutional IRB standards) by emailing the corresponding author.

## AI

A generative AI language model (ChatGPT, OpenAI) was used to assist with phrasing, grammar, and organization during manuscript drafting. All scientific content, interpretations, and conclusions were developed by the authors. All AI-assisted text was reviewed, edited, and verified for accuracy by the authors.
