# Correction: Applying the ADAPT-ITT framework to adapt a Lifestyle Redesign® occupational therapy intervention for diabetic foot ulcer self-management

**DOI:** 10.1093/tbm/ibag059

**Published:** 2026-09-05

This is a correction to: Stacey L Schepens Niemiec, Lauren Styer, Jasmine Chapman, Elliot Myong, Ryan Moshtael, Christopher Lopez, Jocelyn Arteaga, Elaine Wong, Diana Motta Morales, Neel Kumar, Tze-Woei Tan, Applying the ADAPT-ITT framework to adapt a Lifestyle Redesign® occupational therapy intervention for diabetic foot ulcer self-management, *Translational Behavioral Medicine*, Volume 16, Issue 1, 2026, ibag030, https://doi.org/10.1093/tbm/ibag030

Text in the title has been reformatted to reflect the formal name and branding of the intervention.

