# Supplementary material for: Applying the ADAPT-ITT framework to adapt a lifestyle Redesign® occupational therapy intervention for diabetic foot ulcer self-management
Source: Transl Behav Med. 2026 Jun 2;16(1):ibag030. doi: 10.1093/tbm/ibag030 (PMC13228136; doi:10.1093/tbm/ibag030)
Supplement: ibag030_Supplementary_Data [file ibag030_supplementary_data.zip › Supp Table 1 theater characteristics 2026 0421.pdf]

Supplemental Table 1. Participant characteristics and theater testing session composition (*N*=14).

Panel A: Participant characteristics (full sample, *N*=14)

Characteristic	Combined Groups[a]
Age (years)	55 [40,61][b]
Sex: Male	10 (71%)
Race	
American Indian/Alaska Native	1 (7%)
Asian	3 (21%)
White	7 (50%)
Prefer not to disclose/Unknown	3 (21%)
Ethnicity	
Latino/Hispanic	3 (21%)
non-Hispanic/non-Latino	3 (21%)
Prefer not to disclose/Unknown	3 (21%)

Panel B: Theater testing session composition and simulation exposure by topic.

	Group 1 (*n*=3)	Group 2 (*n*=4)	Group 3 (*n*=3)	Group 4 (*n*=2)	Group 5 (*n*=2)
Stakeholder type	1. Vascular surgeon<br>2. Patient<br>3. Patient	1. Podiatrist<br>2. Podiatrist<br>3. Nurse<br>4. Patient	1. Physician<br>2. Podiatrist<br>3. Podiatrist	1. Patient<br>2. Patient	1. Prosthetist/ Orthotist<br>2. Prosthetist/ Orthotist
Lifestyle Redesign® simulation videos shown	1. Foot self-care<br>2. Diabetes diet and nutrition	1. Mental health and diabetes management<br>2. Preparing for healthcare visits	1. Energy management and physical activity<br>2. Preparing for healthcare visits	1. Foot self-care<br>2. Mental health and diabetes management	1. Foot self-care<br>2. Energy management and physical activity

[a]Groups include a small focus group and triadic and dyadic interview sessions
[b]Values are median [interquartile range] or *n* (%)
