# Supplementary material for: Applying the ADAPT-ITT framework to adapt a lifestyle Redesign® occupational therapy intervention for diabetic foot ulcer self-management
Source: Transl Behav Med. 2026 Jun 2;16(1):ibag030. doi: 10.1093/tbm/ibag030 (PMC13228136; doi:10.1093/tbm/ibag030)
Supplement: ibag030_Supplementary_Data [file ibag030_supplementary_data.zip › Supp Table 2 Fidelity Checklist 2026 0409.pdf]

Supplemental Table 2. Fidelity monitoring checklist for the adapted diabetic foot ulcer-specific Lifestyle Redesign® occupational therapy intervention.

Intervention Fidelity Checklist

**Record ID**:	**Date session occurred**:	**Date session reviewed**:

**Session type reviewed**:<br>☐ Treatment session<br>☐ Initial evaluation<br>☐ Progress evaluation<br>☐ Discharge evaluation	**Fidelity assessment data source**:<br>☐ Real-time observation of session<br>☐ Session audio/video recording<br>☐ Session transcript<br>☐ Observer fieldnotes from session

Fidelity Flag Definition: A fidelity flag is triggered when a section does not meet minimum fidelity criteria.

Section 1: *Lifestyle Redesign* Core Characteristics

Characteristic	Rating (0–2)	Notes/Supporting Detail<br>*(e.g., quotes, transcript timestamp, fieldnote excerpt, description of observed behavior)*
**Focus on orchestrating daily activities, lifestyle patterns, or daily routines**	(2) Fully present<br>(1) Partially present<br>(0) Not present	
**Applicable to lifestyle-related challenges**	(2) Fully present<br>(1) Partially present<br>(0) Not present	
**Therapist–client collaboration evident**	(2) Fully present<br>(1) Partially present<br>(0) Not present	
**Aimed at improving health and well-being outcomes**	(2) Fully present<br>(1) Partially present<br>(0) Not present	
**Delivered by an LR-OT trained practitioner**	(2) Yes<br>(0) No	

Scoring Criteria: All characteristics are expected to be present, at least partially, in a session. Sum items to obtain a score.

Section 1 Score (0–10): ___/10

Interpretation and Action Guidance:
8–10 = High fidelity | No action needed
6–7 = Moderate fidelity | Monitor for patterns across sessions
<6 = Low fidelity | Fidelity flag triggered – review session and provide feedback as appropriate

<table>
<tr><th colspan="3">Section 2: Lifestyle Redesign Core Domains</th></tr>
<tr><th>Domain</th><th>Addressed (✔)</th><th>Notes/Supporting Detail
(e.g., quotes, transcript timestamp, fieldnote excerpt, description of observed behavior)</th></tr>
<tr><td>Core Domain 1: Meaningful occupations, habits, and routines</td><td>☐ Addressed</td><td></td></tr>
<tr><td>Core Domain 2: Occupations relevant to wellness and/or chronic condition management</td><td>☐ Addressed</td><td></td></tr>
<tr><td>Core Domain 3: Mental health and psychosocial well-being</td><td>☐ Addressed</td><td></td></tr>
<tr><td>Core Domain 4: Environment and context</td><td>☐ Addressed</td><td></td></tr>
<tr><td>Core Domain 5: Healthcare access and advocacy</td><td>☐ Addressed</td><td></td></tr>
<tr><td colspan="3">Scoring Criteria: Count the number of domains marked as addressed. At least one domain must be addressed to meet fidelity expectations.
(1) One or more domains addressed
(0) No domains addressed</td></tr>
<tr><td colspan="3">Section 2 Score (0–1): ___/1</td></tr>
<tr><td colspan="3">Interpretation and Action Guidance:
1 = Meets fidelity expectations | No action needed
0 = Does not meet fidelity expectations | Fidelity flag triggered – review session and provide feedback as appropriate</td></tr>
</table>

Section 3: *Lifestyle Redesign* Core Techniques

Technique	Identified (✔)	Notes/Supporting Detail<br>*(e.g., quotes, transcript timestamp, fieldnote excerpt, description of observed behavior)*
**Patient education**	☐ Identified	
**Occupational self-analysis**	☐ Identified	
**Narrative reasoning**	☐ Identified	
**Occupational orchestration and engagement**	☐ Identified	
**Activity analysis**	☐ Identified	
**Problem solving**	☐ Identified	
**Autonomy-enhancing communication**	☐ Identified	

Scoring Criteria: Count the number of techniques identified. At least two techniques are expected for adequate fidelity.
(2) Two or more techniques identified
(1) One technique identified
(0) No techniques identified

Section 3 Score (0–2):___/2

Interpretation and Action Guidance:
(2) Meets fidelity expectations | No action needed
(1) Below fidelity expectations | Fidelity flag triggered – review session and monitor for patterns across sessions
(0) Does not meet fidelity expectations | Fidelity flag triggered – review session and provide feedback as appropriate

Section 4: Diabetic Foot Ulcer (DFU) Management		
**Criterion**	**Rating**	**Notes/Supporting Detail**<br>*(e.g., quotes, transcript timestamp, fieldnote excerpt, description of observed behavior)*
**Strength of alignment to DFU management as relevant to client:** *Session clearly focused on DFU management as relevant to client such as footcare routines, offloading device use, or glucose control.*	(2) Strong DFU management alignment<br>(1) Weak or implicit connection to DFU management<br>(0) No clear DFU management relevance	
**Scoring Criteria:** Some variability expected depending on session focus.		
**Section 4 Score (0–2):___**/2		
**Interpretation and Action Guidance:**<br>2 = Meets fidelity expectations | No action needed<br>1 = Below fidelity expectations | Monitor for patterns across sessions<br>0 = Does not meet fidelity expectations | Fidelity flag triggered – review session and provide feedback as appropriate		

Grand Total Score<br>(Sum of Sections 1–4)	
**Total Score (Sections 1–4):___**/15	
**Overall Interpretation**	**Action Guidance**
13–15 High fidelity	No action needed
10–12 Moderate fidelity	Monitor for patterns; review if persistent
<10 Low fidelity	Review session and provide tailored feedback or booster training
**Flagging and Action Protocol**<br>A fidelity flag is triggered when any section does not meet minimum fidelity criteria.<br>Flagged sessions should be reviewed regardless of total score.<br>If ≥20% of reviewed sessions per therapist are flagged (e.g., 2 out of 10 sessions), initiate targeted feedback and coaching.	

Note: This document reflects the general structure of the fidelity checklist; specific definitions derived from proprietary *Lifestyle Redesign* certification materials are intentionally excluded. Fidelity scoring criteria and thresholds are specific to this study.
