# Supplementary material for: Applying the ADAPT-ITT framework to adapt a lifestyle Redesign® occupational therapy intervention for diabetic foot ulcer self-management
Source: Transl Behav Med. 2026 Jun 2;16(1):ibag030. doi: 10.1093/tbm/ibag030 (PMC13228136; doi:10.1093/tbm/ibag030)
Supplement: ibag030_Supplementary_Data [file ibag030_supplementary_data.zip › XXXSupplement_Data.zip!/Supp Table 2 Fidelity Checklist 2026 0409.docx]

**Supplemental Table 2.** Fidelity monitoring checklist for the adapted diabetic foot ulcer-specific Lifestyle Redesign® occupational therapy intervention.

| **Intervention Fidelity Checklist** | | | |
| --- | --- | --- | --- |
| **Record ID**: | **Date session occurred:** | | **Date session reviewed:** |
| **Session type reviewed:**   - Treatment session - Initial evaluation - Progress evaluation - Discharge evaluation | | **Fidelity assessment data source:**  Real-time observation of session  Session audio/video recording  Session transcript  Observer fieldnotes from session | |
| **Fidelity Flag Definition**: A fidelity flag is triggered when a section does not meet minimum fidelity criteria. | | | |

| **Section 1: *Lifestyle Redesign* Core Characteristics** | | |
| --- | --- | --- |
| **Characteristic** | **Rating (0–2)** | **Notes/Supporting Detail**  *(e.g., quotes, transcript timestamp, fieldnote excerpt, description of observed behavior)* |
| **Focus on orchestrating daily activities, lifestyle patterns, or daily routines** | (2) Fully present  (1) Partially present  (0) Not present |  |
| **Applicable to lifestyle-related challenges** | (2) Fully present  (1) Partially present  (0) Not present |  |
| **Therapist–client collaboration evident** | (2) Fully present  (1) Partially present  (0) Not present |  |
| **Aimed at improving health and well-being outcomes** | (2) Fully present  (1) Partially present  (0) Not present |  |
| **Delivered by an LR-OT trained practitioner** | (2) Yes  (0) No |  |
| **Scoring Criteria:** All characteristics are expected to be present, at least partially, in a session. Sum items to obtain a score. | | |
| **Section 1 Score (0–10):** ___/10 | | |
| **Interpretation and Action Guidance:**  8–10 = High fidelity \| No action needed  6–7 = Moderate fidelity \| Monitor for patterns across sessions  <6 = Low fidelity \| Fidelity flag triggered – review session and provide feedback as appropriate | | |

| **Section 2: *Lifestyle Redesign* Core Domains** | | |
| --- | --- | --- |
| **Domain** | **Addressed (✔)** | **Notes/Supporting Detail**  *(e.g., quotes, transcript timestamp, fieldnote excerpt, description of observed behavior)* |
| **Core Domain 1:** Meaningful occupations, habits, and routines | Addressed |  |
| **Core Domain 2:** Occupations relevant to wellness and/or chronic condition management | Addressed |  |
| **Core Domain 3:** Mental health and psychosocial well-being | Addressed |  |
| **Core Domain 4:** Environment and context | Addressed |  |
| **Core Domain 5:** Healthcare access and advocacy | Addressed |  |
| **Scoring Criteria:** Count the number of domains marked as addressed. At least one domain must be addressed to meet fidelity expectations.  (1) One or more domains addressed  (0) No domains addressed | | |
| **Section 2 Score (0–1):** ___/1 | | |
| **Interpretation and Action Guidance:**  1 = Meets fidelity expectations \| No action needed  0 = Does not meet fidelity expectations \| Fidelity flag triggered – review session and provide feedback as appropriate | | |

| **Section 3: *Lifestyle Redesign* Core Techniques** | | |
| --- | --- | --- |
| **Technique** | **Identified (✔)** | **Notes/Supporting Detail**  *(e.g., quotes, transcript timestamp, fieldnote excerpt, description of observed behavior)* |
| **Patient education** | Identified |  |
| **Occupational self-analysis** | Identified |  |
| **Narrative reasoning** | Identified |  |
| **Occupational orchestration and engagement** | Identified |  |
| **Activity analysis** | Identified |  |
| **Problem solving** | Identified |  |
| **Autonomy-enhancing communication** | Identified |  |
| **Scoring Criteria:** Count the number of techniques identified. At least two techniques are expected for adequate fidelity.  (2) Two or more techniques identified  (1) One technique identified  (0) No techniques identified | | |
| **Section 3 Score (0–2):**___/2 | | |
| **Interpretation and Action Guidance:**  (2) Meets fidelity expectations \| No action needed  (1) Below fidelity expectations \| Fidelity flag triggered – review session and monitor for patterns across sessions  (0) Does not meet fidelity expectations \| Fidelity flag triggered – review session and provide feedback as appropriate | | |

| **Section 4: Diabetic Foot Ulcer (DFU) Management** | | |
| --- | --- | --- |
| **Criterion** | **Rating** | **Notes/Supporting Detail**  *(e.g., quotes, transcript timestamp, fieldnote excerpt, description of observed behavior)* |
| **Strength of alignment to DFU management as relevant to client:** *Session clearly focused on DFU management as relevant to client such as footcare routines, offloading device use, or glucose control.* | (2) Strong DFU management alignment  (1) Weak or implicit connection to DFU management  (0) No clear DFU management relevance |  |
|  |  |  |
| **Scoring Criteria:** Some variability expected depending on session focus. | | |
| **Section 4 Score (0–2):**___/2 | | |
| **Interpretation and Action Guidance:**  2 = Meets fidelity expectations \| No action needed  1 = Below fidelity expectations \| Monitor for patterns across sessions  0 = Does not meet fidelity expectations \| Fidelity flag triggered – review session and provide feedback as appropriate | | |

| **Grand Total Score**  (Sum of Sections 1–4) | |
| --- | --- |
| **Total Score (Sections 1–4):**___/15 | |
| **Overall Interpretation** | **Action Guidance** |
| 13–15 High fidelity | No action needed |
| 10–12 Moderate fidelity | Monitor for patterns; review if persistent |
| <10 Low fidelity | Review session and provide tailored feedback or booster training |
| **Flagging and Action Protocol**  A fidelity flag is triggered when any section does not meet minimum fidelity criteria.  Flagged sessions should be reviewed regardless of total score.  If ≥20% of reviewed sessions per therapist are flagged (e.g., 2 out of 10 sessions), initiate targeted feedback and coaching. | |

*Note*: This document reflects the general structure of the fidelity checklist; specific definitions derived from proprietary *Lifestyle Redesign* certification materials are intentionally excluded. Fidelity scoring criteria and thresholds are specific to this study.
