# Supplementary material for: Applying the ADAPT-ITT framework to adapt a lifestyle Redesign® occupational therapy intervention for diabetic foot ulcer self-management
Source: Transl Behav Med. 2026 Jun 2;16(1):ibag030. doi: 10.1093/tbm/ibag030 (PMC13228136; doi:10.1093/tbm/ibag030)
Supplement: ibag030_Supplementary_Data [file ibag030_supplementary_data.zip › XXXSupplement_Data.zip!/Supp Table 1 theater characteristics 2026 0421.docx]

| **Supplemental Table 1.** Participant characteristics and theater testing session composition (*N*=14). | | | | | | |
| --- | --- | --- | --- | --- | --- | --- |
| **Panel A:** Participant characteristics (full sample, *N*=14) | | | | | | |
| **Characteristic** | | | **Combined Groups**^a^ | | | |
| Age (years) | | | 55 [40,61]^b^ | | | |
| Sex: Male | | | 10 (71%) | | | |
| Race | | |  | | | |
| American Indian/Alaska Native | | | 1 (7%) | | | |
| Asian | | | 3 (21%) | | | |
| White | | | 7 (50%) | | | |
| Prefer not to disclose/Unknown | | | 3 (21%) | | | |
| Ethnicity | | |  | | | |
| Latino/Hispanic | | | 3 (21%) | | | |
| non-Hispanic/non-Latino | | | 3 (21%) | | | |
| Prefer not to disclose/Unknown | | | 3 (21%) | | | |
| **Panel B:** Theater testing session composition and simulation exposure by topic. | | | | | | |
|  | **Group 1**  (*n*=3) | **Group 2**  (*n*=4) | | **Group 3**  (*n*=3) | **Group 4**  (*n*=2) | **Group 5**  (*n*=2) |
| Stakeholder type | 1. Vascular surgeon 2. Patient 3. Patient | 1. Podiatrist 2. Podiatrist 3. Nurse 4. Patient | | 1. Physician 2. Podiatrist 3. Podiatrist | 1. Patient 2. Patient | 1. Prosthetist/ Orthotist 2. Prosthetist/ Orthotist |
| Lifestyle Redesign® simulation videos shown | 1. Foot self-care 2. Diabetes diet and nutrition | 1. Mental health and diabetes management 2. Preparing for healthcare visits | | 1. Energy management and physical activity 2. Preparing for healthcare visits | 1. Foot self-care 2. Mental health and diabetes management | 1. Foot self-care 2. Energy management and physical activity |
| ^a^Groups include a small focus group and triadic and dyadic interview sessions  ^b^Values are median [interquartile range] or *n* (%) | | | | | | |
